# Digital Transformation Needs Trustworthy Artificial Intelligence

**DOI:** 10.1016/j.mcpdig.2023.05.007

**Published:** 2023-06-30

**Authors:** Andreas Holzinger

**Affiliations:** Human-Centered AI Lab, University of Natural Resources and Life Sciences, Vienna, Austria and Medical University of Graz, Austria

As the Editor-in-Chief, Francisco Lopez-Jimenez,^1^ very impressively pointed out in his first editorial that there is no way to stop the digital transformation, whether we like it or not, much like the steam engine or the electric current. And the comparison is apt—data is our oil today, and artificial intelligence (AI) is the new electricity because AI is now nearly everywhere. When AI successes, such as the current chat generative pretrained transformer-4 (ChatGPT4), are discussed in daily newspapers, it is safe to say that we are not only living in a new AI spring but already in an AI summer. In fact, ChatGPT is a good example. It shows what modern machine learning methods are capable of. However, it also very clearly shows the limitations.^2^ After the initial enthusiasm, disillusionment sets in, and then the question arises as to how these machines can be used trustworthily in medicine. And that brings us to our topic.

Perhaps the most important topic of AI in medicine, but also in many other areas of application, is trust*.* The new work by Farah et al^3^ shows very impressively and convincingly that trust in AI-based medical devices depends on transparency (interpretability and explainability of the results) and ethics (in the sense of trustworthiness and regulation).^4^ The authors, after identifying the 3 main evaluation criteria for AI-based medical devices according to the Health Technology Assessment guidelines, provided a set of tools and methods to help understand how and why Machine Learning algorithms work and what predictions they make. This is of particular interest now and in the future because digital transformation (with AI as a vehicle to get there) is expected to change medicine permanently and help doctors diagnose and treat diseases, but also facilitate workflows in daily life, such as the time-consuming but mandatory medical documentation.

Ideally, by reducing the routine tasks, the time freed up should be used for the following things that only human experts and not machines can do now:

### Creative Thinking

Although AI is able to generate art, music, or even writing, it is to date not able to create something similar to what humans can do; AI simply lacks the intuition and creativity of a human mind.^5^

### Complex Decision Making

AI can make decisions on the basis of factual data quickly and in parallel, however, AI lacks the ability to consider the wider context, social norms, ethical considerations, and genuinely human personal values, which are often essential in complex decision making.^6^

### Empathy and Emotional Intelligence

Although AI is technically able to recognize emotions using sensors,^7^ it is neither able to experience nor express emotions, nor can AI interpret them in any kind of human-like manner. Empathy and emotional intelligence are essential in many professions, such as counseling, or social work—and in medicine!

### Physical Dexterity

Although AI-driven robots and cyber-physical systems are able to perform many physical tasks, they cannot match the dexterity and flexibility of human hands, particularly in fine and delicate tasks, such as operation (the operative robots we have are not robots in the technical definitions, they are manipulators—the knowledge comes from the human surgeon).^8^

### Understanding and Navigating Social Contexts

AI may understand language and recognize faces—technically, but it lacks the ability to comprehend social contexts and nuances, such as cultural norms, body language, and context-dependent meanings. This ability is crucial in many professions, such as diplomacy, law, or teaching, and in medicine.

### Ethical and Moral Judgment

AI can follow ethical rules and guidelines but cannot make ethical or moral judgments or weigh different values and interests in complex situations that require critical thinking and reflection. This capacity is particularly important in professions such as law, politics, and in medicine.

### Contextual Flexibility

Although AI can learn from large data sets (ChatGPT is a good example) and is able to identify patterns, it lacks the flexibility to adapt to new contexts or situations quickly. Human experts have the ability to apply their expertise to completely novel situations, use their intuition, and adjust their approach to suit the specific needs of the situation. Humans are awesome—they have common sense (in German: Hausverstand).

AI can definitely help find new solutions to the most pressing challenges facing our health care system, but for all its benefits, the widespread adoption of AI technologies also holds large and unforeseen potential for novel and unforeseen threats.^9^ Therefore, it is essential to ensure that AI is developed with these potential threats in mind and that the safety, re-traceability, transparency, explicability, validity, and verifiability of AI applications in every medical application are ensured. And here we come full circle. To ensure this interpretability in a broader sense, Farah et al^3^ have noted that metrics and methods for “explainable AI” need to be combined with ethical and legal analyses, and that acceptable standards for explainability are always context-dependent and depend on the risks of the clinical scenario.

Raising awareness of these concepts is crucial to their widespread adoption and answering ethical questions. We must succeed in this in the future. We must collectively take a synergistic approach to human-centered AI internationally to enable humans to take control of AI by aligning AI with human intelligence, human values, ethical, and legal requirements to ensure secure and safe human-AI interactions—robustness and trust are the necessary ingredients.^10^ This can be done by using the human-in-the-loop approach to interactively include the human into the AI pipeline.

## Potential Competing Interests

The author declares that there are no competing financial interests or personal relationships that could have appeared to influence the work reported in this paper. This work does not raise any ethical issues.
